# Health-related quality of life in end-stage renal disease patients: the effects of starting dialysis in the first year after the transition period

**DOI:** 10.1007/s11255-018-1845-6

**Published:** 2018-03-26

**Authors:** Natascha J. H. Broers, Remy J. H. Martens, Bernard Canaud, Tom Cornelis, Tom Dejagere, Nanda M. P. Diederen, Marc M. H. Hermans, Constantijn J. A. M. Konings, Frank Stifft, Joris J. J. M. Wirtz, Karel M. L. Leunissen, Frank M. van der Sande, Jeroen P. Kooman

**Affiliations:** 1 Division of Nephrology, Department of Internal Medicine, Maastricht University Medical Center+, PO Box 5800, 6202 AZ Maastricht, The Netherlands; 2 NUTRIM School of Nutrition and Translational Research in Metabolism, Maastricht University, Maastricht, The Netherlands; 3 Fresenius Medical Care GmbH, Bad Homburg, Germany; 4 Department of Nephrology, Jessa Hospital, Hasselt, Belgium; 5 Department of Internal Medicine, Division of Nephrology, Viecuri Medical Center, Venlo, The Netherlands; 6 Department of Internal Medicine, Division of Nephrology, Catharina Hospital Eindhoven, Eindhoven, The Netherlands; 7 Department of Internal Medicine, Division of Nephrology, Zuyderland Medical Center, Sittard-Geleen, The Netherlands; 8 Department of Internal Medicine, Division of Nephrology, Laurentius Hospital Roermond, Roermond, The Netherlands

**Keywords:** Health-related quality of life, Physical Activity, End-stage renal disease, Dialysis

## Abstract

**Background/aims:**

Prevalent dialysis patients have low scores of health-related quality of life (HRQOL) which are associated with increased risk of hospitalization and mortality. Also in CKD-5 non-dialysis patients, HRQOL scores seem to be lower as compared with the general population. This study firstly aimed to compare HRQOL between CKD-5 non-dialysis and prevalent dialysis patients in a cross-sectional analysis and to assess longitudinal changes over 1 year after the dialysis initiation. Secondly, the correlation between HRQOL and physical activity (PA) was explored.

**Methods:**

Cross-sectional 44 CKD-5 non-dialysis, 29 prevalent dialysis, and 20 healthy controls were included. HRQOL was measured by Short Form-36 questionnaires to measure physical and mental domains of health expressed by the physical component summary (PCS) and mental component summary (MCS) scores. PA was measured by a SenseWear™ pro3. Longitudinally, HRQOL was assessed in 38 CKD-5 non-dialysis patients (who were also part of the cross-sectional analysis), before dialysis initiation until 1 year after dialysis initiation.

**Results:**

PCS scores were significantly lower both in CKD-5 non-dialysis patients and in prevalent dialysis patients as compared with healthy controls (*p* < 0.001). MCS scores were significantly lower in both CKD-5 non-dialysis patients (*p* = 0.003), and in dialysis patients (*p* = 0.022), as compared with healthy controls. HRQOL scores did not change significantly from the CKD-5 non-dialysis phase into the first year after dialysis initiation. PA was significantly related to PCS in both CKD-5 non-dialysis patients (*r* = 0.580; *p* < 0.001), and dialysis patients (*r* = 0.476; *p* = 0.009).

**Conclusions:**

HRQOL is already low in the CKD-5 non-dialysis phase. In the first year after dialysis initiation, HRQOL did not change significantly. Given the correlation between PCS score and PA, physical activity programs may be potential tools to improve HRQOL in both CKD-5 non-dialysis as well as in prevalent dialysis patients.

**Electronic supplementary material:**

The online version of this article (10.1007/s11255-018-1845-6) contains supplementary material, which is available to authorized users.

## Introduction

Numerous studies already showed lower health-related quality of life (HRQOL) scores in dialysis patients as compared with the general population [1–3]. Low HRQOL scores were shown to be predictive of hospitalization and mortality in this patient group [4–6]. Nevertheless, in most studies, general trends at different time periods with a varying patient cohort were compared [1, 7], and only a limited amount of studies followed HRQOL in dialysis patients over time in the same patients [8, 9]. A previous study of our group showed the prognostic value of changes in HRQOL over time in maintenance hemodialysis (HD) patients, but did not include the stage 5 chronic kidney disease (CKD-5) non-dialysis phase [4]. Few prospective studies have focused on the effects of starting dialysis treatment on HRQOL [9, 10], despite the fact that in patients with end-stage renal disease (ESRD) the transition from the CKD-5 non-dialysis phase to the start of dialysis is a major life event [11–13]. Theoretically, HRQOL may decrease following the start of dialysis due to the invasiveness of the therapy, or might be improved due to the partial resolution of uremic symptoms. Previous studies showed a reduction in HRQOL in patients with CKD-5 in the non-dialysis phase as compared with the general population [10, 14, 15], but a comparative study between CKD-5 non-dialysis and prevalent dialysis patients, or a longitudinal study following the start of dialysis before dialysis initiation, has not yet been performed.

In interpreting alterations in HRQOL, it is important to identify potentially modifiable factors. Previously, we observed a relation between nutritional parameters and changes in HRQOL [4]. Recently, a larger international cohort study observed a significant relation between physical activity (PA) and HRQOL in maintenance dialysis patients [16]. However, in this study, PA was assessed by self-reported scales and not by objective measurements. In a previous study of our group, we observed a significant increase in walking speed 6 months after the start of dialysis as compared with the CKD-5 non-dialysis phase [17]. Correlations between changes in HRQOL and changes in PA following the start of dialysis have not been studied yet.

Aims of this study were firstly to compare HRQOL between healthy controls, CKD-5 non-dialysis, and prevalent dialysis patients, and secondly, to assess changes from the CKD-5 non-dialysis phase until 1 year after the start of dialysis and thirdly, to assess correlations between HRQOL and PA parameters, both in a cross-sectional and longitudinal design.

## Materials and methods

**Fig. 1:**
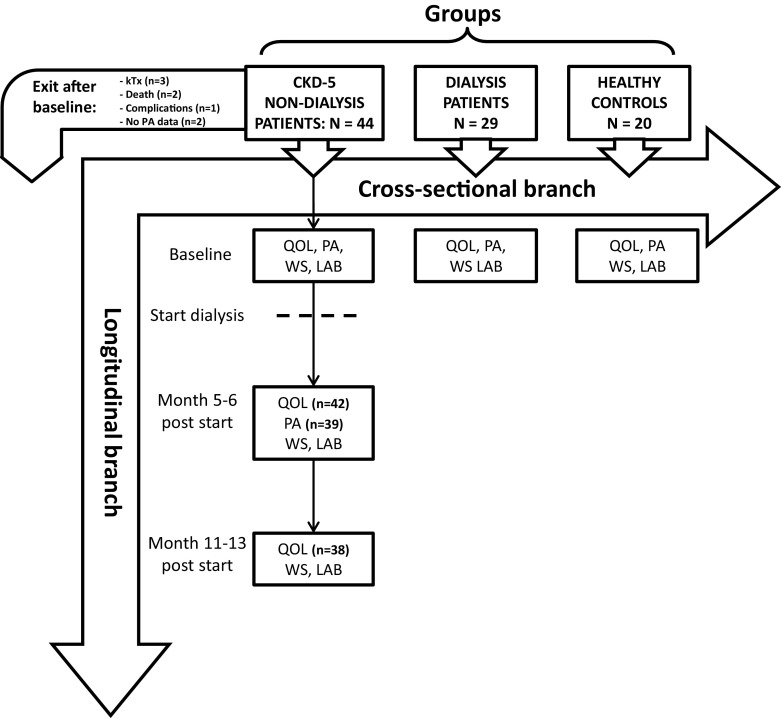
Study design. *kTx* kidney transplantation, *PA* physical activity, *CKD* chronic kidney disease, *N* number of patients, *QOL* quality of life, *WS* walking speed, *LAB* laboratory parameters

This study consisted of a cross-sectional part and a longitudinal part (Fig. 1). The methodology (as described below) has been described previously [17].

For the cross-sectional analyses, we included 73 patients, 44 CKD-5 non-dialysis and 29 prevalent dialysis patients (dialysis vintage 3.6 ± 3.2 years) as well as 20 healthy controls. Patients were recruited from the following dialysis centers in the South East of the Netherlands and North East of Belgium: Maastricht University Medical Center+, Catharina Hospital Eindhoven, Viecuri Hospital Venlo, Zuyderland Medical Center Sittard, St Laurentius Hospital Roermond and Jessa Hospital Hasselt. CKD-5 non-dialysis patients were ESRD patients starting with dialysis within 1 month. (Measurements were performed maximum 4 weeks prior to the first dialysis session.) Prevalent dialysis patients were treated with hemodialysis (HD) or peritoneal dialysis (PD) treatment for at least 12 months.

The longitudinal analyses with regard to HRQOL scores included 38 of the CKD-5 non-dialysis patients (who were also part of the cross-sectional analysis) in the transitional phase for whom 12-month follow-up data after dialysis initiation were available (for PA analyses 39 patients were included). Measurements were performed before the start of dialysis treatment (within 1 month before the first dialysis session), 5–6 months, and 11–13 months after dialysis initiation by the same methods as used for the cross-sectional part. In addition, PA was only measured before the start of dialysis and 5–6 months after the start of dialysis, no data were available 1 year after the start of dialysis due to the study design.

Exclusion criteria for CKD-5 non-dialysis patients were: an acute start of dialysis treatment, active symptomatic coronary artery disease or cardiac failure New York Heart Association (NYHA) class III or IV, active malignancies, active infections, and inability to provide informed consent. For walking test measurements, physical disability was measured. (Patients had to be able to walk without help.) Exclusion criteria for prevalent dialysis patients were similar.

Healthy controls were non-diabetic, non-smokers, and not hypertensive (systolic blood pressure < 170 mmHg and/or diastolic blood pressure < 100 mmHg) and were recruited via advertisements at the university hospital.

Patients, as well as healthy controls, were asked to be in a fasting state during the measurements, except for the PA measurements.

Written informed consent was obtained from each patient prior to participation. The study was approved by the Ethical Committee (NL33129.068.10, NL35039.068.10) and the Hospital Board of the Maastricht University Medical Center+.

### Quality of life measurements

Short Form-36 (SF-36) version 1 questionnaires were filled out to measure physical component summary (PCS) scores for the physical domains of health, and mental component summary (MCS) for the mental domains of health. The SF-36 questionnaire is the most used tool to measure HRQOL in the field of nephrology worldwide [18] and is a reliable and valid instrument for use in both general population surveys and in studies of chronic disease populations in the Netherlands [19]. The SF-36 is a multi-purpose, short-form health survey which includes 36 items. These 36 items provide a measure of physical and mental health items ranging from 0 (“worst possible health”) to 100 (“best possible health”). The 36 items can be subdivided into eight subscales known as physical functioning (PF), role-physical (RP), bodily pain (BP), general health (GH), vitality (VT), social functioning (SF), role-emotional (RE), and mental health (MH). These eight subscales were summarized in two summary scores known as: PCS score and a MCS score. Questionnaires were scored by scoring algorithms of Ware et al. [20, 21]. All scales were normalized via* t*-score transformation (mean, 50 ± 10 [SD]) to make it comparable to the general population and other patient groups with specific disease states [21, 22].

### Physical activity measurements

#### Number of steps

All participants wore a SenseWear™ pro 3 armband (BodyMedia^®^, Pittsburg, PA) to measure PA parameter number of steps for 2 days (CKD-5 non-dialysis patients: 2.01 ± 0.41 days, mean on-body time: 94.9%; prevalent dialysis patients: 2.30 ± 0.73 days, mean on-body time: 96.7%; controls: 2.07 ± 0.54 days, mean on-body time: 96.1%), which is considered to be sufficient to obtain data with regard to daily PA [23, 24]. The mean of the total on-body time was calculated (expressed as number of steps per 24 h) to include both the dialysis and non-dialysis day. No differentiation was made between data collected on week or weekend days for all participants.

#### Four-meter walking test

A 4-m walking test was conducted to determine walking speed (m/sec) by covering a distance of 4 m. Several studies confirmed the validity and sensitivity of this widely used test for determining walking speed [25–28] and physical performance in ESRD patients [29].

### Biochemical parameters

Albumin, hemoglobin (HB), dialysis adequacy (Kt/V), and β2-microglobulin were measured or determined during routine patient laboratory measurements (Table 1). The residual glomerular filtration rate (GFR_residual_) was estimated with the use of β2-microglobulin as described by Vilar et al. [30].

**Table 1:** Patient characteristics cross-sectional analysis

	CKD-5 non-dialysis patients	Dialysis patients	Healthy controls
Number of patients	44	29	20
Male (%)	75.0	69.0	65.0
HD/PD	–	21*/8	–
Age (years)	61.3 ± 12.0	58.17 ± 14.65	59.65 ± 14.10
Height (cm)	173.8 ± 9.3	171.59 ± 9.57	174.75 ± 11.36
Weight (kg)	79.2 ± 17.3	82.75 ± 15.31	76.68 ± 15.80
BMI (kg/m^2^)	26.0 ± 4.1	28.06 ± 4.45	24.86 ± 3.41
Albumin (g/L)	35.34 ± 5.18 (*n* = 36)	40.24 ± 3.55 (*n* = 25)	40.24 ± 2.30
Hemoglobin (mmol/L/g/dL)	6.7 ± 0.9/10.8 ± 1.4 (*n* = 39)	6.9 ± 0.7/11.1 ± 1.2 (*n* = 28)	–
Kt/V (HD/PD)	–	1.54 ± 0.39/2.17 ± 0.74	–
eGFR (ml/min/1.73 m^2^)	13.8 ± 5.5 (*n* = 36)	–	72.59 ± 11.07
Origin of end-stage renal disease			
Diabetic nephropathy (%)	4.5	20.7	–
Polycystic kidney disease (%)	27.3	17.2	
Nephrosclerosis (%)	15.9	6.9	
Hypertensive nephropathy (%)	9.1	10.3	
Nephrotic syndrome (%)	11.4	3.4	
Unknown (%)	11.4	13.8	
Other (%)	20.5	27.6	
Diabetes mellitus (%)	15.9	41.4	–
Cardiovascular disease (%)	34.1	37.9	–
Risk of mortality by Davies index			
Low risk (%)	47.4	41.4	–
Medium risk (%)	43.2	37.9	
High risk (%)	9.1	20.7	
History of prior KTx (%)	22.7	31.0	0.0
SBP (mmHg)	146.0 ± 20.9	152.4 ± 26.5	138.0 ± 13.4
DBP (mmHg)	83.1 ± 12.9	80.8 ± 12.4	82.3 ± 6.9

Data are given in mean ± SD. *HD* hemodialysis, *PD* peritoneal dialysis, *BMI* body mass index, *eGFR* estimated glomerular filtration rate, *KTx* kidney transplantation, *SBP* systolic blood pressure, *DBP* diastolic blood pressure * All HD patients have arteriovenous (AV) fistulas

### Comorbidity score

Comorbidity index was determined for each patient based on the comorbidity checklist by the Davies comorbidity index scoring system [31]. Patients were divided into three risk groups: low, medium, and high risk of mortality. The Davies comorbidity index is commonly used for ESRD patients [31–33].

### Longitudinal analysis

Changes in parameters of HRQOL were measured in 38 CKD-5 non-dialysis patients before the start of dialysis (within 1 month before start), 5–6 months after starting dialysis, and 11–13 months after the start of dialysis by the same methods as for the cross-sectional part. Changes in parameters of PA were measured in 39 CKD-5 non-dialysis patients before the start of dialysis and 5–6 months after the start of dialysis due to the study design. It was measured by the same methods as for the cross-sectional part. (Data are previously described elsewhere [17]).

### Statistical analysis

Data are expressed as mean ± SD or median [25th–75th percentile], unless indicated otherwise.

For the cross-sectional analyses, differences in the categorical variables were assessed using Chi-square tests. Differences in the SF-36 summary scores (PCS and MCS) between groups were assessed by linear regression analyses. Differences in the individual subscales of the SF-36 were examined with Mann–Whitney *U* tests as scores of these subscales were not normally distributed. In additional analyses, we adjusted the between-group differences in PCS and MCS scores for differences in the distribution of age, gender, and diabetes status with the use of multivariable regression analyses. As PA was lower in both CKD-5 non-dialysis patients and prevalent dialysis patients, as was already described in our previous study [17], we subsequently examined correlations between these PA parameters and HRQOL scores, as well as correlations between biochemical parameters and HRQOL scores with Spearman’s rank correlation coefficients.

Changes in the CKD-5 non-dialysis patient group during the first 12 months after dialysis initiation were examined using Friedman tests, as most variables were not normally distributed. Each analysis was based on all available data. Differences in change between dialysis modalities were examined with Friedman tests. Correlations between changes in PA parameters and changes in HRQOL scores were assessed with Spearman’s rank correlation coefficients.

All statistical analyses were performed with IBM SPSS Statistics for Windows, version 24 (IBM Corp. Armonk, NY, USA). *p* values ≤ 0.05 were considered to be statistically significant.

## Results

### Patient characteristics

Patient characteristics for the cross-sectional part are summarized in Table 1. Patient characteristics for the patients participating in the longitudinal part were similar to baseline characteristics for both the longitudinal measurements of HRQOL as well as PA (Supplementary Tables 1a and 1b).

### Quality of Life outcomes

**Fig. 2:**
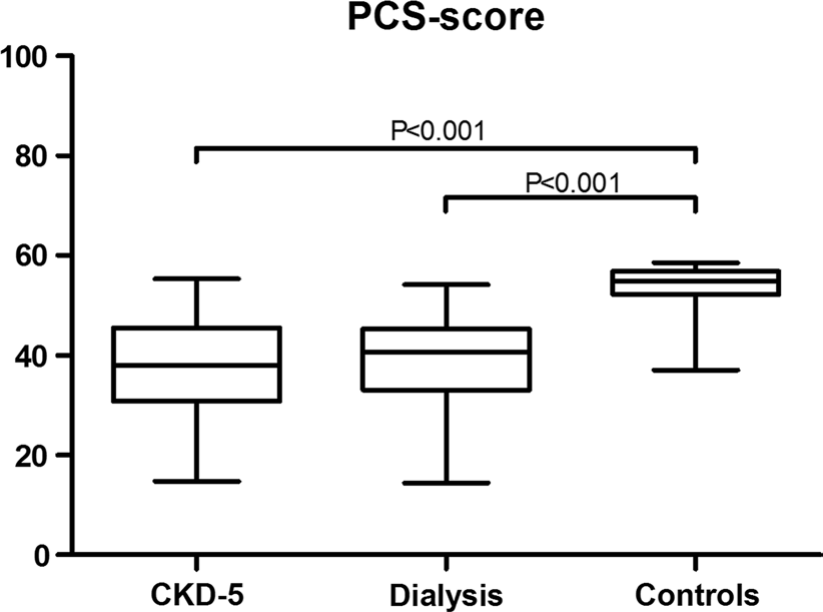
Physical component summary (PCS) scores. *CKD-5* stage 5 chronic kidney disease

In linear regression analyses, the difference in PCS scores as compared with controls was (beta [95% CI]) − 15.6 [− 20.4, − 10.8] for CKD-5 non-dialysis patients (*p* < 0.001), and − 15.5 [− 20.7, − 10.3] for the prevalent dialysis patients (*p* < 0.001). PCS scores did not statistically significantly differ between CKD-5 non-dialysis and prevalent dialysis patients (*p* = 0.955) (Fig. 2).

**Fig. 3:**
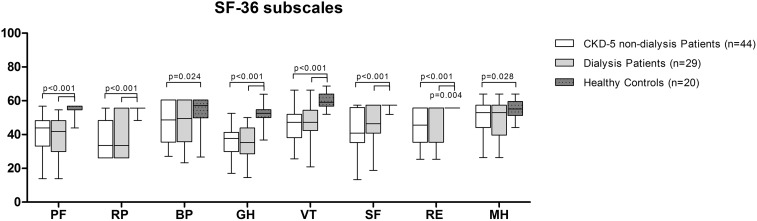
Subscale domains SF-36. *PF* physical functioning, *RP* role-physical, *BP* bodily pain, *GH* general health, *VT* vitality, *SF* social functioning, *RE* role-emotional, *MH* mental health

Subsequent nonparametric analyses showed that the scores on the subscales which correlate with PCS scores (i.e. PF, RP, GH, and VT) were statistically significantly lower in CKD-5 non-dialysis patients, and prevalent in dialysis patients as compared with healthy controls, except for BP, which was only statistically significantly lower in CKD-5 non-dialysis patients (Fig. 3).

**Fig. 4:**
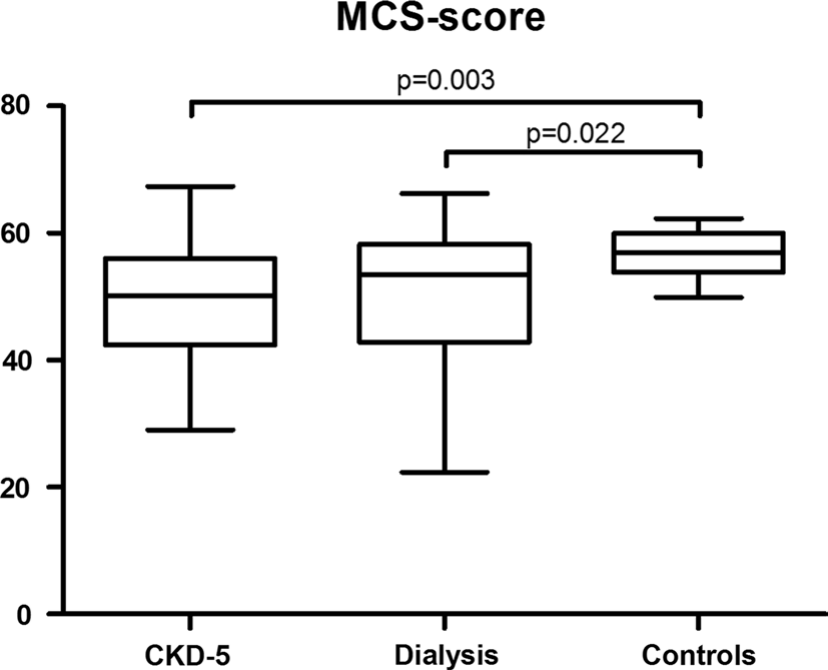
Mental component summary (MCS) scores. *CKD-5* stage 5 chronic kidney disease

In linear regression analyses, the difference in MCS scores as compared with controls was (beta [95% CI]) − 7.3 [− 12.0, − 2.6] for CKD-5 non-dialysis patients (*p* = 0.003), and − 6.0 [− 11.1, − 0.9] for the prevalent dialysis patients (*p* = 0.022). MCS scores did not statistically significantly differ between CKD-5 non-dialysis and prevalent dialysis patients (*p* = 0.540) (Fig. 4).

Subsequent nonparametric analyses showed that the scores on the subscales which correlate with MCS scores (i.e., RE, SF, VT and GH) were statistically significantly lower in CKD-5 non-dialysis patients, and prevalent dialysis patients as compared with healthy controls, except for MH which was only statistically significantly lower in CKD-5 non-dialysis patients (Fig. 3).

After adjustment for age, gender, and diabetes prevalence, outcomes were not materially changed (data not shown).

### Physical activity outcomes

As already described in a previous study of our group [17], the median number of steps was statistically significantly lower in both CKD-5 non-dialysis patients (5435.5 [3212.8–7384.3]) and prevalent dialysis patients (3994.5 [1993.5–6712.8]) as compared with healthy controls (11,062.0 [7687.0–13,839.0]) (*p* < 0.001).

Also walking speed was statistically significantly lower in CKD-5 non-dialysis patients (median 1.4 [1.2–1.8] m/s) as compared with healthy controls (1.8 [1.7–2.0] m/s) (*p* = 0.017), but not as compared with prevalent dialysis patients (1.5 [1.2–1.9] m/s) (*p* = 0.699). After adjustment for age, gender, and diabetes, prevalence outcomes were not materially changed (data not shown) [17].

### Longitudinal outcomes HRQOL

**Fig. 5:**
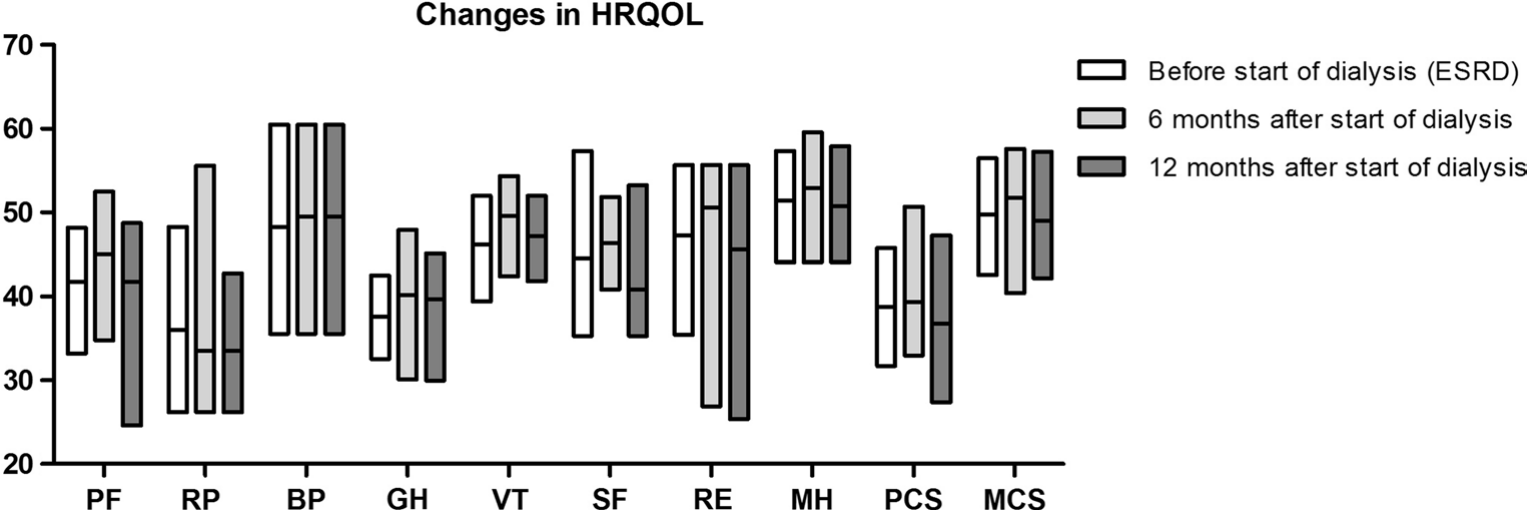
Changes in HRQOL (subscales). Boxes represent median, 25th percentile and 75th percentile. *HRQOL* health-related quality of life, *PF* physical functioning, *RP* role-physical, *BP* bodily pain, *GH* general health, *VT* vitality, *SF* social functioning, *RE* role-emotional, *MH* mental health, *PCS* physical component summary, *MCS* mental component summary, *ESRD* end-stage renal disease

In the first year after the start of dialysis, PCS scores did not change statistically significantly (*p* = 0.275). Also, additional analyses of the subscales for PCS score, PF, RP, BP, GH, and VT did not show statistically significant changes over time (Table 2, Fig. 5).

**Table 2:** Longitudinal analyses HRQOL scales

Scale	Visit	*n* = 38	*P* value
Physical functioning	Before start dialysis	43.9 [33.2–48.2]	0.089
	6 months after start dialysis	45.0 [34.8–52.5]	
	12 months after start dialysis	41.8 [24.6–48.7]	
Role-physical	Before start	33.6 [26.2–48.3]	0.804
	6 months after start dialysis	33.6 [26.2–55.6]	
	12 months after start dialysis	33.6 [26.2–44.8]	
Bodily pain	Before start	48.6 [35.6–60.5]	0.771
	6 months after start dialysis	49.5 [35.6–60.5]	
	12 months after start dialysis	49.5 [35.6–60.5]	
General health	Before start	37.7 [32.6–42.5]	0.271
	6 months after start dialysis	40.2 [30.1–48.0]	
	12 months after start dialysis	39.7 [30.0–45.1]	
Vitality	Before start	47.2 [39.4–52.0]	0.394
	6 months after start dialysis	49.6 [42.4–54.4]	
	12 months after start dialysis	47.2 [41.8–52.0]	
Social functioning	Before start	40.8 [35.3–57.4]	0.644
	6 months after start dialysis	46.3 [40.8–51.9]	
	12 months after start dialysis	40.8 [35.3–53.2]	
Role-emotional	Before start	50.6 [35.5–55.7]	0.573
	6 months after start dialysis	50.6 [26.9–55.7]	
	12 months after start dialysis	45.6 [25.4–55.7]	
Mental health	Before start	52.9 [44.1–57.3]	0.694
	6 months after start dialysis	52.9 [44.1–59.6]	
	12 months after start dialysis	50.7 [44.1–57.9]	
Physical component summary score	Before start	38.9[31.7–45.8]	0.275
	6 months after start dialysis	39.3 [33.0–50.7]	
	12 months after start dialysis	36.8 [27.3–47.3]	
Mental component summary score	Before start	50.1 [42.5–56.6]	0.900
	6 months after start dialysis	51.7 [40.4–57.6]	
	12 months after start dialysis	49.0 [42.2–57.3]	

Data are given in median [25th and 75th percentile]

MCS scores did not change statistically significantly in the first year after the start of dialysis (*p* = 0.900). The same held true for the additional analyses of the subscales for MCS score; MH, RE, SF, VT, and GH did not show statistically significant changes over time (Table 2, Fig. 5).

When separated by dialysis modality (19 patients started with HD and 19 patients started with PD), no statistically significant changes over time were found for the different dialysis modalities.

In PD patients, median PCS score before the start of dialyses was 37.8 [32.5–44.6], after 5–6 months of dialysis, median PCS score was 36.8 [32.2–45.8], and 1 year after the start of dialysis median PCS score was 33.5 [29.1–42.6] (*p* = 0.504). In HD patients, median PCS score before the start of dialyses was 40.5 [31.7–48.6], after 5–6 months of dialysis median PCS score was 42.8 [33.1–51.6], and 1 year after the start of dialysis, median PCS score was 45.5 [24.0–50.0] (*p* = 0.532).

In PD patients, median MCS score before the start of dialyses was 48.1 [42.3–58.4], after 5–6 months of dialysis median MCS score was 51.6 [37.5–56.3], and 1 year after the start of dialysis, median MCS score was 52.2 [42.3–58.2] (*p* = 0.810). In HD patients, median MCS score before the start of dialyses was 51.0 [45.6–54.7], after 5–6 months of dialysis median MCS score was 54.6 [40.6–57.7], and 1 year after the start of dialysis, median MCS score was 47.3 [42.0–57.0] (*p* = 1.000).

### Longitudinal outcomes of physical activity

As already described in our previous study, no statistically significant changes over time in the first 6 months after starting dialysis were found for median number of steps, which changed from 5747.0 [3137.0–7808.0] to 5486.0 [3892.0–8452.0]; *p* = 0.052 (*n* = 39) [17]. As also described in the previous study, walking speed statistically significantly increased in the first 6 months after the start of dialysis from median 1.4 [1.2–1.8] to 1.7 [1.5–2.0] m/s (*p* = 0.050) (*n* = 34) [17]. However, an additional analysis in 29 patients for whom 1-year follow-up data were available in the present study with regard to walking speed showed no statistically significant differences in walking speed 1 year after the start of dialysis (*p* = 0.161): walking speed was, respectively, (1.4 [1.3–1.9] m/s) before the start of dialysis, (1.7 [1.6–1.9] m/s) 5–6 months after the start of dialysis, and (1.5 [1.3–1.9] m/s) 1 year after the start of dialysis.

### Correlations between PA parameters and HRQOL scores

**Fig. 6:**
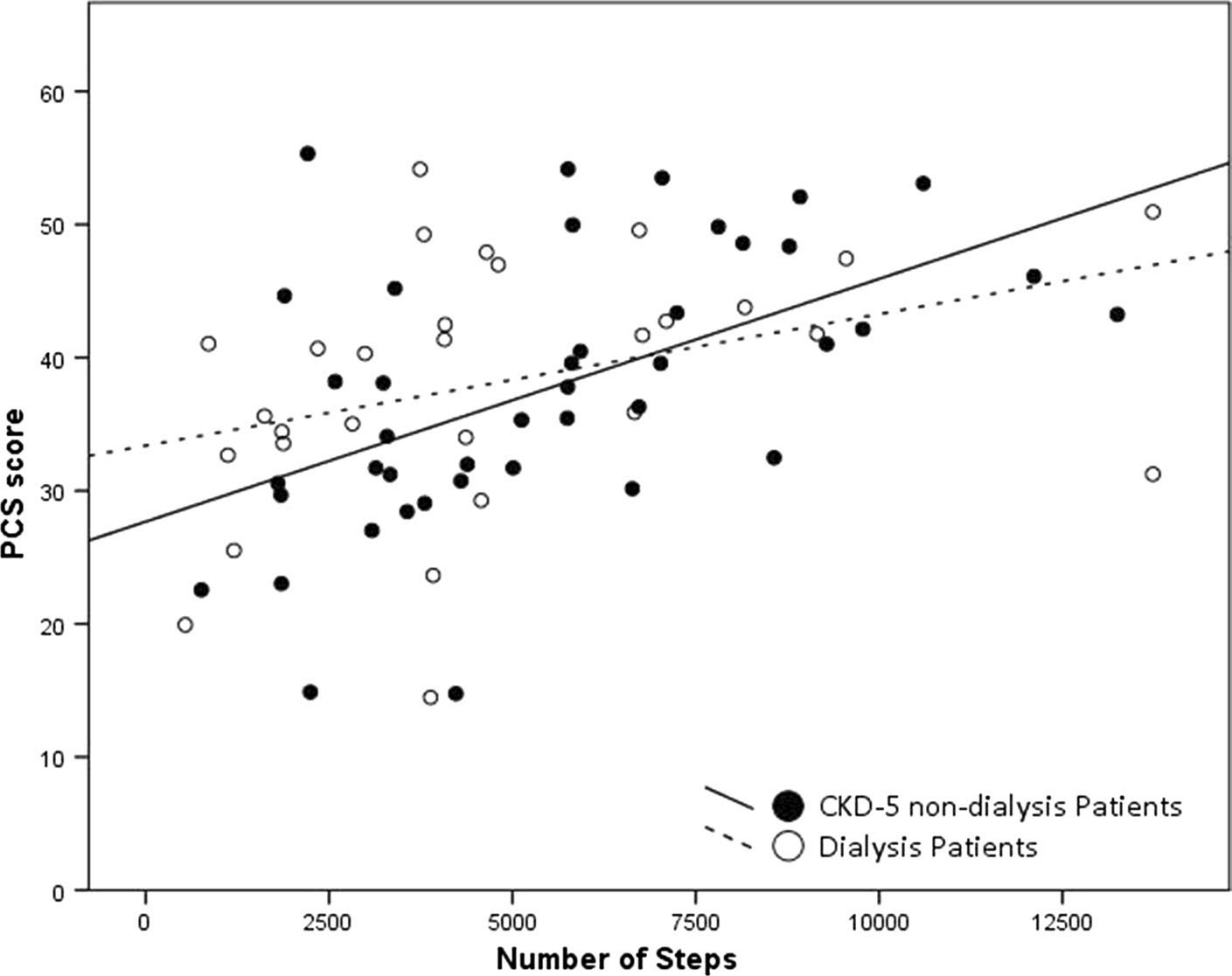
Correlation between physical component summary (PCS) score and number of steps in both CKD-5 non-dialysis (*r*_s_ = 0.580; *p* < 0.001) and dialysis patients (*r*_s_ = 0.476; *p* = 0.009)

Correlations were found for number of steps and PCS scores in both CKD-5 non-dialysis (*r*_s_ = 0.580; *p* < 0.001) as well as in prevalent dialysis patients (*r*_s_ = 0.476; *p* = 0.009) (Fig. 6). No correlations were found with MCS scores: CKD-5 non-dialysis patients (*r*_s_ = 0.041; *p* = 0.797) and prevalent dialysis patients (*r*_s_ = 0.1158; *p* = 0.542). The subscales PF, RP, VT, and SF, which correlate with the PCS score, were correlated with number of steps in CKD-5 non-dialysis patients: PF (*r*_s_ = 0.570; *p* < 0.001), RP (*r*_s_ = 0.486; *p* = 0.001), VT (*r*_s_ = 0.381; *p* = 0.013), and SF (*r*_s_ = 0.319; *p* = 0.040). In prevalent dialysis patients only in subscale PF a correlation was found (*r*_s_ = 0.548; *p* = 0.002).

**Fig. 7:**
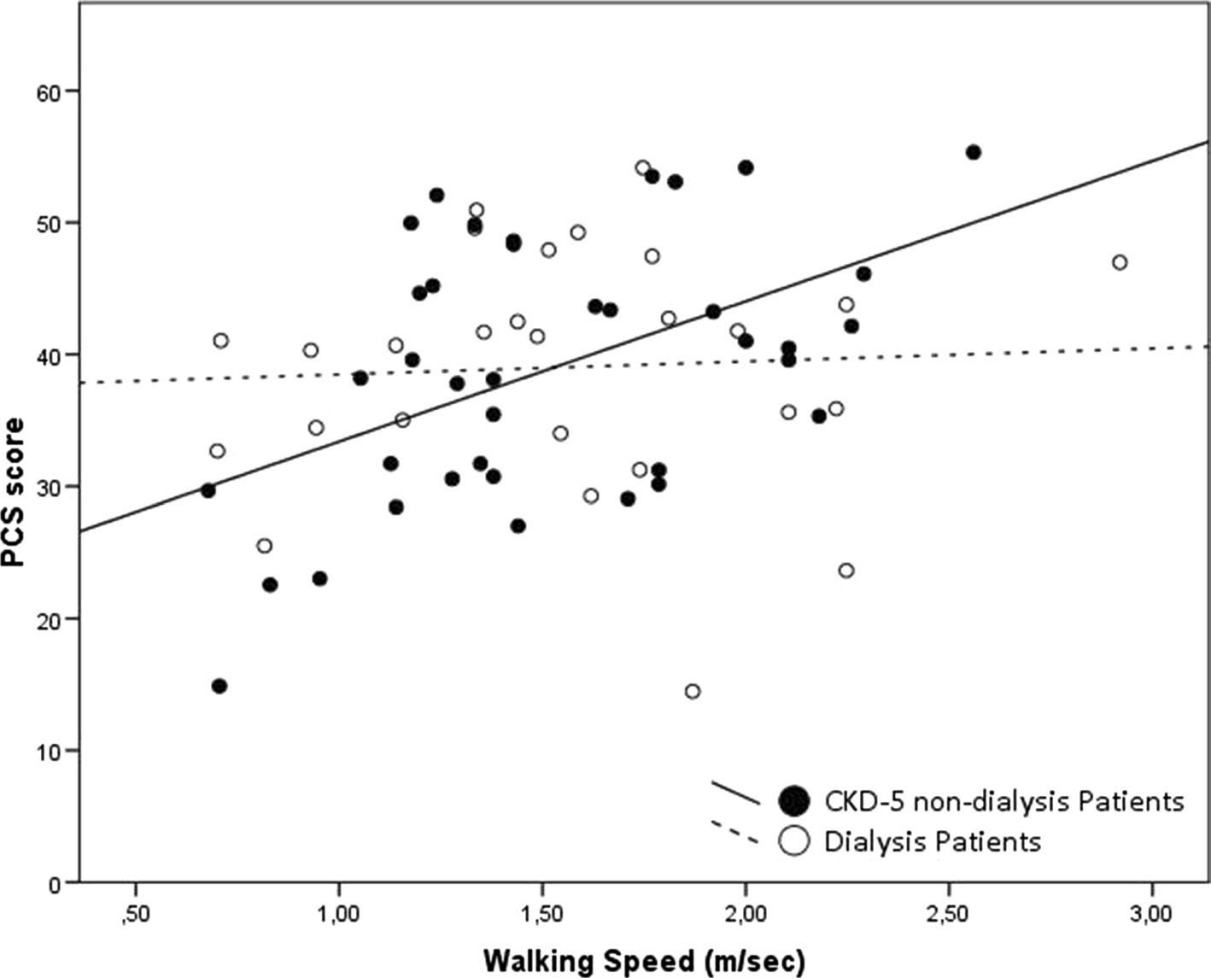
Correlation between physical component summary (PCS) score and walking speed in both CKD-5 non-dialysis (*r*_s_ = 0.415; *p* = 0.010) and dialysis patients (*r*_s_ = 0.097; *p* = 0.630)

Furthermore, correlations were found for walking speed and PCS scores in CKD-5 non-dialysis patients (*r*_s_ = 0.415; *p* = 0.010) but not in prevalent dialysis patients (*r*_s_ = 0.097; *p* = 0.630) (Fig. 7). No correlations were found with MCS scores: CKD-5 non-dialysis patients (*r*_s_ = -0.007; *p* = 0.967) and prevalent dialysis patients (*r*_s_ = 0.063; *p* = 0.756). The subscales PF, VT, and SF, which correlate with the PCS score, were correlated with walking speed in CKD-5 non-dialysis patients: PF (*r*_s_ = 0.537; *p* < 0.001), RP (*r*_s_ = 0.331; *p* = 0.042), VT (*r*_s_ = 0.351; *p* = 0.031), and SF (*r*_s_ = 0.441; *p* = 0.006). In prevalent dialysis patients, no correlations for subscales were found.

No correlations were found for delta number of steps and both delta PCS and delta MCS scores in the first 6 months after the start of dialysis. The same held true for delta walking speed and both delta PCS and delta MCS scores after both the first 6 months as well as the first year after the start of dialysis.

### Correlations between biochemical parameters and HRQOL scores

No correlations were found for HB and PCS scores in both the CKD-5 non-dialysis group (*r*_s_ = − 0.061; *p* = 0.710), as well as in the dialysis group (*r*_s_ = − 0.098; *p* = 0.621). Also no correlations were found for HB and MCS scores in both the CKD-5 non-dialysis group (*r*_s_ = 0.089; *p* = 0.590), as well as in the dialysis group (*r*_s_ = 0.128; *p* = 0.518).

The same held true for albumin and PCS scores: CKD-5 non-dialysis group (*r*_s_ = 0.293; *p* = 0.083) and dialysis group (*r*_s_ = 0.190; *p* = 0.362). Here also, no correlations were found for albumin and MCS scores in both the CKD-5 non-dialysis group (*r*_s_ = 0.226; *p* = 0.186), as well as in the dialysis group (*r*_s_ = − 0.154; *p* = 0.463).

## Discussion

In brief, this study firstly showed a reduction in the physical domains of the SF-36 HRQOL scores in both CKD-5 non-dialysis and prevalent dialysis patients, compared with age-matched healthy controls, without significant differences between both patient groups. The summary score of the mental domains of the SF-36 scale (MCS) was significantly lower in both CKD-5 non-dialysis patients and prevalent dialysis patients as compared with controls, without significant differences between CKD-5 non-dialysis and prevalent dialysis patients. Secondly, in the longitudinal analysis, no significant changes in HRQOL were observed in one of the most critical periods for ESRD patients: the phase from CKD-5 non-dialysis phase until 1 year after the start of dialysis [11–13], neither in the physical, nor in the mental components. Lastly, PA parameters were significantly associated with PCS scores and components of the PCS scale of the SF-36, which was most pronounced for the PF component. No correlations were found for changes in PA (delta number of steps/delta walking speed) and changes in both PCS and MCS scores (delta PCS/delta MCS).

The cross-sectional analysis showed PCS score values below norm-based scores in CKD-5 non-dialysis patients as well as in prevalent dialysis patients and significantly lower scores as compared with healthy controls, suggesting reduced HRQOL in this patient group. In particular, subscales physical functioning, role-physical, and general health, which are highly associated with the physical component [21], were low as compared with norm-based values. Previous research in a large US dialysis cohort showed a comparable reduction in PCS and MCS scores of the SF-36 in both incident and prevalent dialysis patients [4]. Our findings are in line with previous studies also, whereby in incident patients [34], and in both CKD (stage 2–5) patients [35] as well as in HD patients [2, 36], lower HRQOL scores were observed. However, to the best of our knowledge, no comparative study has been performed between CKD-5 non-dialysis and prevalent dialysis patients. Interestingly, these findings are not only common in ESRD patients, but also in other populations with chronic diseases, such as chronic obstructive pulmonary disease (COPD) [37, 38] and heart failure [39], low HRQOL scores are observed.

In the longitudinal analysis, no significant changes in PCS or MCS scores were observed in the first year after starting dialysis, in which patients were followed from the CKD-5 non-dialysis phase. This is remarkable given the fact that the start of dialysis is a critical phase in ESRD patients [11, 12] with both phenotypic and pathophysiologic changes [13], an increased risk of mortality [40, 41], and a significantly decreased functional status, which was observed in elderly nursing home patients after dialysis initiation [42]. We should acknowledge that our patient group may consist of a selected group, as all patients were recruited from the CKD-5 non-dialysis out-patient clinic. Nevertheless, the severely impaired HRQOL in the CKD-5 non-dialysis phase underscores the need for strategies to improve HRQOL already in earlier stages of CKD [43].

One of these potentially modifiable factors includes PA. We observed a significant relation between objectively measured PA parameters and physical domains of the PCS. A study of Painter et al. showed increased self-reported physical scale and PCS scores in patients receiving exercise training [44]. A more recent study of Lopes et al. also showed that higher self-reported aerobic physical activity level was associated with better physical HRQOL (higher PCS scores) in HD patients [16]. The results of our study thus confirm findings from earlier research although in our study objective measures were used to assess PA by the SenseWear Pro3™. Next to that, the correlation between changes in HRQOL and changes in PA was longitudinally assessed over a 1-year time period. Despite the correlation between PA and PCS scores in the cross-sectional analysis, contrastingly, no correlations were found for changes in PA parameters and changes in PCS scores over a period of time of 12 months. This might be due to the fact that changes in PA following the start of dialysis were not significant in the overall group and relatively small in individual patients, or due to the possibility that SF-36 scores might not be sensitive enough to detect dialysis-specific changes in HRQOL.

Several limitations of the study deserve consideration; first, due to the small study sample, in particular, the longitudinal analyses are underpowered, as reflected by the wide interquartile ranges in these analyses. Nevertheless, our cross-sectional analyses showed a large clinically relevant difference between both patient groups and the healthy control group, even when in the analyses the lower bound of the 95% confidence interval was considered. Furthermore, our findings are comparable with earlier studies [2, 4, 5], which supports the outcomes in this present study. Second, both PD and HD patients were included, with apparently little differences between both groups. It is, however, important to realize that this study focused on the effects of starting dialysis treatment per se and not on differences in dialysis modalities, for which a larger sample size would be required. However, a previous study did not conclude notable differences in HRQOL between HD and PD treatment also [9]. Third, no matching procedure in group formation of the CKD-5 non-dialysis group and the prevalent dialysis group was conducted, which increased the possibility of bias. However, in additional analyses, we adjusted the between-group differences for differences in the distribution of age, gender, and diabetes status. In addition, outcomes were not materially changed. Fourth, only the SF-36 version 1 questionnaire was used to measure HRQOL instead of the Kidney Disease Quality of Life-36 (KDQOL-36) which is especially designed for patients with ESRD. Nevertheless, the SF-36 is the most widely used tool to measure HRQOL in the field of nephrology worldwide [18], and scores were normalized which made it comparable to the general population (healthy controls) [21]. Last, due to the fact that we have included relatively young and possibly less frail controls and patients, there might be a possibility of selection bias. In addition, out of the 44 patients who started dialysis, 2 patients died after the first 6 months of dialysis.

In conclusion, the physical domain of HRQOL (as measured by PCS of the SF-36) is already severely decreased in the CKD-5 non-dialysis phase. Additionally, in the first year after the start of dialysis treatment, HRQOL scores did not change significantly, suggesting no major effect of the start of dialysis in our ESRD patients, in whom the reduced HRQOL appears to be primarily related to the renal disease per se. Next to HRQOL also PA is low in both CKD-5 non-dialysis as well as prevalent dialysis patients. Given the correlation between the physical domain of health (PCS) and PA, the importance of physical activity programs should be underscored and strongly encouraged in both CKD-5 non-dialysis as well as in dialysis patients in order to increase the physical domains of HRQOL in these patients groups.

## Electronic supplementary material

Below is the link to the electronic supplementary material.

Supplementary material 1 (DOC 58 kb)
