# Supplementary material for: Health-related quality of life in end-stage renal disease patients: the effects of starting dialysis in the first year after the transition period
Source: Int Urol Nephrol. 2018 Mar 26;50(6):1131–42. doi: 10.1007/s11255-018-1845-6 (PMC5986848; doi:10.1007/s11255-018-1845-6)
Supplement: Supplementary file 1 — Supplementary material 1 (DOC 58 kb) [file 11255_2018_1845_MOESM1_ESM.doc]

**Journal: International Urology and Nephrology**

**Health-related quality of life in end-stage renal disease patients: the effects of starting dialysis in the first year after the transition period.**

Natascha J.H. Broers ^1,2^ Remy J.H. Martens ^1,2^ Bernard Canaud ^3^ Tom Cornelis ^4^ Tom Dejagere ^4^ Nanda M.P. Diederen ^1^ Marc M.H. Hermans ^5^ Constantijn J.A.M. Konings ^6^ Frank Stifft ^7^ Joris J.J.M. Wirtz ^8^ Karel M.L. Leunissen ^1^ Frank M. van der Sande ^1^ Jeroen P. Kooman ^1,2^

1) Department of Internal Medicine, Division of Nephrology, Maastricht University Medical Center+

2) NUTRIM School of Nutrition and Translational Research in Metabolism, Maastricht University, Maastricht

3) Fresenius Medical Care D GmbH, Bad Homburg, Germany

4) Department of Nephrology, Jessa Hospital, Hasselt, Belgium;

5) Department of Internal Medicine, Division of Nephrology, Viecuri Medical Center, Venlo

6) Department of Internal Medicine, Division of Nephrology, Catharina Hospital Eindhoven, Eindhoven , The Netherlands

7) Department of Internal Medicine, Division of Nephrology, Zuyderland Medical Center Sittard-Geleen

8) Department of Internal Medicine, Division of Nephrology, Laurentius Hospital Roermond, Roermond

E-mail address: natascha.broers@mumc.nl

**Supplementary Tables**

**Table 1a.** Baseline patient characteristics longitudinal analysis of HRQOL.

| **Number of Patients** | 38 |
| --- | --- |
| **Male (%)** | 71.1 |
| **HD*/PD^#^** | 19/19 |
| **Age (years)** | 60.6±12.1 |
| **Height (cm)** | 172.9±9.6 |
| **Weight (kg)** | 78.0±17.5 |
| **BMI (kg/m^2^)** | 25.8±4.2 |
| **Albumin (g/L) (*n=31*)** | 36.1±3.9 |
| **Hemoglobin (mmol/L / g/dL) (*n=35*)** | 6.7±0.9 / 10.8±1.4 |
| **Kt/V (HD (*n=17*) /PD (*n=17*))** | 1.3±0.1 / 2.5±1.0 |
| **eGFR (ml/min/1.73m^2^) (*n=31*)** | 14.3±5.6 |
| **Origin of end-stage renal disease**   - Diabetic nephropathy (%) - Polycystic kidney disease (%) - Nephrosclerosis(%) - Hypertensive nephropathy (%) - Nephrotic syndrome (%) - Unknown (%) - Other (%) | 5.3  26.3  15.8  10.5  10.5  10.5  21.1 |
| **Diabetes Mellitus (%)** | 13.2 |
| **Cardiovascular Disease (%)** | 31.6 |
| **Risk of mortality by Davies index**   - Low risk (%) - Medium risk (%) - High risk (%) | 50.0  42.1  7.9 |
| **History of prior KTx (%)** | 23.7 |
| **SBP (mmHg)** | 147.0±21.5 |
| **DBP (mmHg)** | 84.1±13.5 |

Data are given in mean±SD. HD = hemodialysis, PD = peritoneal dialysis, BMI = body mass index, eGFR = estimated glomerular filtration rate, KTx = kidney transplantation, SBP = systolic blood pressure, DBP = diastolic blood pressure * 1 HD patient had a central venous catheter (CVC) # 2 PD patients switched from PD to HD via CVC after the start of dialysis, 1 HD patient switched from HD to PD after the start of dialysis.

**Table 1b.** Baseline patient characteristics longitudinal analysis of PA**.**

| **Number of Patients** | 39 |
| --- | --- |
| **Male (%)** | 74.4 |
| **HD*/PD^#^** | 18/21 |
| **Age (years)** | 61.7±12.2 |
| **Height (cm)** | 173.5±9.6 |
| **Weight (kg)** | 78.3±17.2 |
| **BMI (kg/m^2^)** | 25.8±4.1 |
| **Albumin (g/L) (*n=32*)** | 35.4±5.4 |
| **Hemoglobin (mmol/L / g/dL) (*n=36*)** | 6.7±0.9 / 10.8±1.4 |
| **Kt/V (HD (*n=16*) /PD (*n=19*))** | 1.3±0.2 / 2.4±1.0 |
| **eGFR (ml/min/1.73m^2^) (*n=32*)** | 14.1±5.6 |
| **Origin of end-stage renal disease**   - Diabetic nephropathy (%) - Polycystic kidney disease (%) - Nephrosclerosis(%) - Hypertensive nephropathy (%) - Nephrotic syndrome (%) - Unknown (%) - Other (%) | 5.1  25.6  17.9  10.3  12.8  10.3  17.9 |
| **Diabetes Mellitus (%)** | 15.4 |
| **Cardiovascular Disease (%)** | 30.8 |
| **Risk of mortality by Davies index**   - Low risk (%) - Medium risk (%) - High risk (%) | 48.7  43.6  7.7 |
| **History of prior KTx (%)** | 23.1 |
| **SBP (mmHg)** | 145.8±21.7 |
| **DBP (mmHg)** | 82.9±13.5 |

Data are given in mean±SD. HD = hemodialysis, PD = peritoneal dialysis, BMI = body mass index, eGFR = estimated glomerular filtration rate, KTx = kidney transplantation, SBP = systolic blood pressure, DBP = diastolic blood pressure * 1 HD patient had a central venous catheter (CVC) # 2 PD patients switched from PD to HD via CVC after the start of dialysis, 1 HD patient switched from HD to PD after the start of dialysis.
